# Parenting of mothers of children in early childhood during the COVID-19 pandemic: qualitative research

**DOI:** 10.1590/0034-7167-2022-0478

**Published:** 2023-07-31

**Authors:** Ricardo Barros Gurgel, Juliane Lima Pereira Da Silva, Estela Maria Leite Meirelles Monteiro, Suzana Lins da Silva, Tereza Rebecca de Melo e Lima, Maria Wanderleya de Lavor Coriolano-Marinus

**Affiliations:** I Universidade Federal de Pernambuco. Recife, Pernambuco, Brazil; II Instituto de Medicina Integral Professor Fernando Figueira. Recife, Pernambuco, Brazil

**Keywords:** Parenting, Social Vulnerability, Child Development, COVID-19 Pandemic, Qualitative Research, Responsabilidad Parental, Vulnerabilidad Social, Desarrollo Infantil, COVID-19, Investigación Cualitativa, Poder Familiar, Vulnerabilidade Social, Desenvolvimento Infantil, COVID-19, Pesquisa Qualitativa

## Abstract

**Objectives:**

to understand practices of mothers of children in early childhood who live in contexts of poverty in the face of the COVID-19 pandemic.

**Methods:**

an exploratory, descriptive and qualitative study. Participants were selected in the community context, composing an intentional sample to be collected through semi-structured online interviews. Data were analyzed inductively and anchored in the Bioecological Model of Human Development.

**Results:**

eight mothers participated in the research. Mothers highlighted pleasure in taking care of their children, although they were overloaded with activities and comprehensive care at the time of the pandemic. Children, in mothers’ perception, showed a higher frequency of challenging behaviors, which may be related to negative parenting practices, such as punishment and physical violence.

**Final Considerations:**

interventions to support parenting become urgent in the face of changes brought about by the COVID-19 pandemic in families living in a context of poverty.

## RESUMO

**Objetivos:**

compreender a parentalidade e práticas parentais de mães de crianças na primeira infância que vivem em contextos de pobreza, diante da pandemia de COVID-19.

**Métodos:**

estudo exploratório, descritivo, abordagem qualitativa. As participantes foram selecionadas no contexto comunitário, compondo uma amostra intencional para a coleta por entrevistas semi-estruturadas na modalidade *online*. Os dados foram analisados de forma indutiva e ancorados na Teoria Bioecológica do Desenvolvimento Humano.

**Resultados:**

oito mães participaram da pesquisa. As mães destacaram prazer em cuidar dos filhos, embora estivessem sobrecarregadas com atividades e cuidado integral no momento da pandemia. As crianças, na percepção das mães, apresentaram maior frequência de comportamentos desafiadores, os quais podem ter relação com práticas parentais negativas, como punição e violência física.

**Considerações Finais:**

intervenções de apoio à parentalidade se tornam urgentes diante de mudanças suscitadas pela pandemia de COVID-19 em famílias que vivem em contexto de pobreza.

## RESUMEN

**Objetivos:**

comprender la crianza y las prácticas de crianza de las madres de niños en la primera infancia que viven en contextos de pobreza, frente a la pandemia de la COVID-19.

**Métodos:**

estudio exploratorio, descriptivo, enfoque cualitativo. Los participantes fueron seleccionados en el contexto comunitario, componiendo una muestra intencional para ser recolectada a través de entrevistas semiestructuradas en la modalidad online. Los datos fueron analizados inductivamente y anclados en la Teoría Bioecológica del Desarrollo Humano.

**Resultados:**

ocho madres participaron de la investigación. Las madres destacaron el gusto por cuidar a sus hijos, aunque estaban sobrecargadas de actividades y atención integral en el momento de la pandemia. Los niños, en la percepción de las madres, mostraron una mayor frecuencia de comportamientos desafiantes, lo que puede estar relacionado con prácticas de crianza negativas, como castigo y violencia física.

**Consideraciones Finales:**

las intervenciones de apoyo a la crianza se vuelven urgentes ante los cambios provocados por la pandemia de COVID-19 en las familias que viven en un contexto de pobreza.

## INTRODUCTION

Parenting is defined as a job whose main object of attention and action is children, given that children cannot grow and thrive without the presence of caregivers. The presence of parents/caregivers is fundamental for education and socialization, being divided by Bornstein into cognitive practices and sensitivity practices. Cognitive practices such as oral storytelling and book reading show positive results in language acquisition and academic performance. Sensitive practices are those that engage children in interpersonal interactions, such as when parents make their children feel loved and valued, through the regulation of affections and emotions that help in intrapersonal components and in the relationship with others^(1, 2)^.

The Bioecological Model^(3)^ explains how human development is influenced by people, the environment and the context and time in which we live. Processes in the microfamily environment in the parent-child relationship are influenced by spaces in the mesosystem, such as school, neighborhood and parents’ work. The COVID-19 pandemic, together with changes in the family microsystem, mesosystem and macrosystem, may have had repercussions on peculiar parenting practices in the relationships between caregivers and children, which may have short- and long-term impacts on the development of children in early childhood (period from zero to six years of age)^(4)^, such as an increase in negative parenting practices, due to increased stress and overload in families that already lived with material and emotional deprivation^(5, 6)^.

Women, as the main caregivers, experienced additional overload in their role, in addition to dealing with the reduction of previously existing support networks, such as day care centers, schools, social assistance services and support from neighbors and family members^(7)^.

A review of the impacts of the COVID-19 pandemic, based on the Nurturing Care framework, with data from 30 countries, reinforces differences in family impact, with greater negative experiences in financially vulnerable families, in addition to disproportionate effects for caregivers of young children, particularly women, those with pre-existing mental illnesses, and families of children with disabilities^(8)^.

Absolute poverty usually refers to a state where income is insufficient to provide the basic necessities necessary for survival (food and shelter for children). Relative poverty is defined as the broader implications of living in poverty, such as the inability to participate or contribute to society on an equal basis due to lack of income^(9)^. Poverty affects a child’s development and educational outcomes from the earliest years of life, directly and indirectly through mediating and moderating processes^(10)^.

The knowledge gap of studies on the subjectivity and perception of caregivers living in poverty contexts about their parental actions in the face of the COVID-19 pandemic was found in a review, with only 8% of qualitative studies on caregiver responsiveness, learning and safety, in a sample of 112 studies, with a predominance of studies in developed countries^(8)^.

This study addresses parenting in the reality of developing countries, such as Brazil, in the context of the COVID-19 pandemic. Scientific data elucidated by qualitative studies can contribute to the formulation and implementation of practical strategies that support caregivers’ and children’s needs in early childhood in an international agenda to support the comprehensive development of children in early childhood.

## OBJECTIVES

To understand practices of mothers of children in early childhood who live in contexts of poverty in the face of the COVID-19 pandemic.

## METHODS

### Ethical aspects

The research complied with Resolutions 466/2012 and 510/2016 of the Brazilian National Health Council and Circular Letter 2/2021/ CONEP/SECNS/MoH, which deals with the regulation of data collection through online interviews during the COVID 19 pandemic. The study was approved by the Research Ethics Committee.

### Theoretical-methodological framework

The study was anchored in Bronfenbrenner’s Bioecological Model of Human Development^(3)^ theoretical framework based on the Person, Process, Context and Time (PPCT) dimensions. The developing People considered were children and caregivers. Processes were the relationships between caregiver and child. Context was the COVID-19 pandemic, containing places of the microsystem (home), mesosystem (community space, neighborhood, school, parents’ work). Time was microtime-immediate perspective, mesotime-frequency and extent that relationships take place, macrotime-historical perspective, which impacts not only children, but other individuals and components of the PPCT model.

### Study design

This is an exploratory, descriptive study with a qualitative approach. Qualitative studies express the study of social relations in relation to the different dimensions of the sphere of life, including the subjective meanings that individuals attribute to their activities and environments. We followed the COnsolidated criteria guide for REporting Qualitative research (COREQ) guidelines^(11, 12)^.

### Methodological procedures

Participants’ consent took place online by signing the Informed Consent Form (ICF) by filling out a form using Google Forms®. Then, the interview was scheduled, which was conducted by means of a video call, through WhatsApp®. The interviews were recorded through Google Meet® in parallel with the video call, with audio recording of the interviews on a computer. The interviews were not recorded via WhatsApp®, as this tool was not available at the time the data were collected.

The interviewers were a master’s student (doctor), a doctoral student (nurse), who received theoretical and practical training on qualitative interviews, tutored by the advisor.

### Study setting

The study was carried out in 2020, in a virtual environment, due to social distancing restrictions resulting from the COVID-19 pandemic. Data were collected online with families registered in a social assistance institution, located in the Várzea neighborhood, Recife-Pernambuco. This institution was chosen because it serves families in poverty (monthly per capita income below R$85.00). The institution assists 165 families in poverty. The institution offers activities to support families as a service to strengthen family ties, support learning activities, recreational activities, sports for children and adolescents, in addition to professional courses for adults, in partnership with other institutions.

During the COVID-19 pandemic, the institution was unable to receive children for face-to-face educational activities.

### Data source

Participants were mothers of children aged between zero and six years, participants of the institution’s WhatsApp® group, in which 150 participants were registered. All caregivers received an invitation to participate in the research, from the expression of interest in individual contact with the researcher in charge. After expressing interest, the main researcher made the digital ICF available and scheduled the day and time, according to participants’ convenience. At this stage, only mothers contacted the researcher.

Sampling was non-probabilistic, intentional. The sample size was based on the data saturation criterion. The data saturation concept was considered to be applicable to the point where no new information emerged during data collection, when the capacity to obtain new information was reached and when additional coding did not appear^(13, 14)^.

### Data collection and organization

Data collection took place through online interviews, which took into account the alignment of this methodology to address a phenomenon that happens in participants’ real life (parental relationships between parents and children)^(15)^. The interview was guided by a script, which included mother, child and family characterization, questions about parenting, in line with the bioecological framework^(3)^, from the PPCT elements. The guiding questions were: how has your interaction with your child been in your daily life? Did the COVID-19 pandemic bring any changes to the family routine? Could you talk about these changes?

What activities does your child most enjoy doing? How do you participate in these activities? What do you usually do when children’s behavior displeases you? What do you do for children’s education and cooperation? What do you hope for the future of your child and your family?

The interviews were recorded and later transcribed. The duration of each interview varied, lasting on average about 40 minutes.

### Data analysis

The framework adopted for the qualitative analysis followed Robert Yin’s assumptions based on the five steps^(16)^: 1) Compilation; 2) Decomposition; 3) Recomposition; 4) Interpretation; 5) Conclusion.

The first author, together with the supervisor, developed the line-by-line coding, creating a book of inductive codes based on the data. Throughout the process, the codes were refined into larger codes, for articulation with the Bioecological Model theoretical framework, subsidizing the elaboration of thematic categories: 1) Child growth and development (subthemes Situations in children’s daily life; Conceptualizing child development); 2) Process: relationships between caregivers and children (subthemes Interactions in care, games and support for learning; Caregiver wear; Needs for changes in maternal self-assessment; Positive and negative parenting practices); 3) Context of the COVID-19 pandemic in the microsystem, mesosystem and exosystem (subthemes Changes in the child; Caregiver wear); 4) Time of care and future perspectives (subthemes Time in the caregiver-child relationship; Future prospects for children).

## RESULTS

The results explore data from interviews with eight participants, characterized as children’s biological mothers. Age ranged from 20 to 40 years, with about eight years of complete study and average monthly income between 1 and 2 minimum wages. Only two of the eight interviewees reported not being primarily responsible for full-time child care. Four participants worked outside the home, in activities such as cleaning, maid and self-employed. Of the eight participants, three lived alone with their children, without support from their fathers. From data analysis, the categorization was carried out, in a constructivist way, both inductively from the data, as well as based on the theoretical framework^(2)^.

Chart 1 presents the Bioecological Model axes, categories, subthemes and examples of speeches.

**Chart 1:** Categorization of results, according to the Bioecological Model axes, category, subtopics and speech examples, Recife, Pernambuco, Brazil, 2022

Bioecological Model axes	Category	Subthemes	Examples of speeches
Person - the developing person	Child growth and development	Situations in children’s daily life	[...] *he eats well, he eats alone, it’s not work.* (I7) [...] *eat more on television, watching.* (I3) [...] *he sleeps well, he sleeps all night* [...]. (I7) *He sleeps in the same bed with me.* (I5) [...] *difficulty sometimes putting him to sleep, sometimes he wants to go late* [...]. (I7)
		Conceptualizing child development	[...] *I think it’s the child learning things, developing things.* (I1) [...] *it’s the child being smart, being smart, being unwound.* (I3)
Process - parental relationships between caregiver and child	Process: relationships between caregivers and children	Interactions in care, games and support for learning	[...] *my role inside the house I think is to take care of them, to do things around the house, to play with them, to teach them things, to do the chores* [...]. (I6) *I like to take care of my children, to pay attention to them.* (I6)
		Caregiver wear	*I don’t really see myself as a mother, I see myself more as a maid, I don’t know, because it’s the same thing all day long, bathing, eating, doing things* [...] *I feel practically like a maid in here.* (I5)
		Needs for changes in maternal self-assessment	*I wanted to learn like this, to have a little more patience, to teach them, to have more patience to teach* [...]. (I5)
		Positive and negative parenting practices	*He is always praised* [...] *it is* [...] *a right thing, a thank you that asks* [...]. (I2) *If I see that it’s not working, I’ll punish her* [...] *I’ll make her sit in the corner, on the chair.* (I4) *I give a little slap; I’m not going to lie.* (I3) *Sometimes I speak ignorantly.* (I4)
Context - changes from the COVID-19 pandemic in the microsystem (family), mesosystem (school, daycare, health service, leisure, parents’ work), exosystem (local and global epidemiological context)	Context of the COVID-19 pandemic in the microsystem, mesosystem and exosystem	Changes in the child	*He is a back teller.* (I6) *He keeps yelling at me.* (I3)
		Caregiver wear	*How often do I feel stressed? It is almost always. Then the person is alone at home, there is that tiredness* [...] *they are also discouraged.* (I5) *What bothers me the most is staying indoors, not being able to go out.* (I6) *What makes me sad? I think the difficulties, right, which are many, because I’m alone to take care of him, it’s a lot of difficulty, you have to manage and there’s also the issue of water, there’s a lot of water missing here.* (I7)
		Division of responsibilities and parental participation	*If it was to change, it would be for the father to pay more attention, but as much as he can, he pays attention, he plays, everything.* (I6) *One cooks lunch, another sweeps the house, another washes the dishes* [...]. (I1) [...] *yes, he participates, he is not his father, but he is very affectionate with him, very affectionate indeed, plays with him, bathes him, helps with food* [...] *he does everything.* (I3)
		Support network of neighbors and family	[...] *yes, a neighbor, as I work, I pay her to stay with him during the day.* (I7) *When he goes to my aunt’s house there’s only him, my aunt and her husband, then when he comes back from there* [...] *he gets very back teller* [...] *she ignores him.* (I6)
		Leisure activities	*We went out a lot, we took them to the beach,* [...] *the family had fun, but with the pandemic we are not going out.* (I6)
		Reduced access to health services	*At my health center they say that they are only doing care in extreme cases* [...] *because of the pandemic* [...] *it’s difficult to make an appointment.* (I2)
		Support for learning and absence from school	[...] *I tell him, “Let’s play school”, then the colors, he knows the colors, what the letters are, these things and she also joins in the game, because it’s a way that I have to activate her brain.* (I2) *In the task I say: come, then I’ll sit with him, but then my patience, my psychology I don’t have anymore* [...]. (I5) [...] *that she can study, that the schools start to return to normal* [...]*, that she can go to school to have a better future.* (I4)
		Prevention measures	*I’m wearing a mask, I have gel alcohol, and everything I bring from the street I spray with alcohol at 70.* (I3)
Time - microtime, mesotime and historical perspective	Time of care and future perspectives	Time in the caregiver-child relationship	[...] *I spend all day now, with the pandemic all day.* (I1) *I work, then I only get home around 6, 6 and a half, then when I get home, I stay with him.* (I7)
		Future prospects for children	[...] *I wanted him to study more, continue his studies, which I didn’t do, right* [...] *get into college, have a profession.* (I7) *I only see my son formed; I only see my son a man with a good heart mainly.* (I2)

### Category 1 – Children’s growth and development

In category 1, everyday situations that influence child growth and development are explored, such as eating, sleeping and resting routines, in addition to caregivers’ understanding of the concept of child development. From the Bioecological Model, the child was considered as an individual in the process of development and their dependence on proximal and distal elements as people and environments that contribute to their full development. Most mothers reported that their children ate well, autonomously, some fed with the support of an adult due to the delay or convenience for caregivers. Most children ate their meals using some electronic device, mainly the television. As for children’s sleep routine, the mothers reported no difficulties, but shared the change to later times and the shared bed. As for the concept of child development, participants conceptualized it as learning and acquiring more complex abilities.

### Category 2 - Process: relationships between caregivers and children

In category 2, caregivers assessed their actions and feelings regarding the parental role and described child care in the sense of educating and teaching them to behave. In the self-assessment of their role, the interviewees shared the overload in domestic activities and with the world of work as aspects that negatively influenced the caregiver-child relationship. The main needs were the desire for parental learning and more patience. Among the positive parenting practices, playing with children, reports of enjoying playing with their children, talking when they had disagreements and praising their children stood out. Among the negative parenting practices, they mentioned the use of punishments, threats and moral and/or physical violence. Father participation was reported with ambiguities. Some mentioned that parents or partners participate in child care, but some pointed out that they could participate more, and others that they share household responsibilities when they are present outside of working hours. Regarding the support network, the interviewees reported having help from children’s grandmothers, aunts and neighbors, but they reported negative interference on the part of these caregivers, which negatively interfered in children’s behavior.

### Category 3 - Context of the COVID-19 pandemic in the microsystem, mesosystem and exosystem

Caregivers mentioning changes in the family’s leisure routine, access to health services and impacts of school closures were highlighted in everyday life. As for the school routine, most mothers complained about the lack of day care centers, closed by the COVID-19 pandemic and the repercussions for both learning, children’s social relationships and increased family burden. Some mothers reported the effort in trying to teach their children school activities, but reported difficulties in supporting the teaching-learning process in establishing routines and regularity. Caregivers also mentioned the main preventive measures against COVID-19 at a time when vaccination actions were not yet available to the population.

### Category 4 - Time of care and future prospects

The concept of time, according to the theoretical framework used, involves the entire dimension of temporality involved in the human being’s development process^(3)^. In the study, we divided this category into two perspectives: one focused on the care time that caregivers dedicate to children and the other on the perspective of this child’s future development. Caregivers who were not active in the world of work mentioned a full-time care routine, particularly in the context of the pandemic. All caregivers mentioned projecting a better future for their children, which could break the entire national cycle of poverty, based on education and moral values.

## DISCUSSION

The results show parental relationships between mothers and their children and the interference of family support systems during adaptive changes from the 2020 COVID-19 pandemic. The worsening of difficulties in the challenges of parenting during the pandemic was identified in families that already lived with financial difficulties, such as behavioral changes in children and increased intra-family stress of caregivers.

The data analyzed, from the bioecological perspective of human development, proposed by Bronfenbrenner^(3)^, when considering the development of children, from biological components and their interrelationship with people and environment, portrays the existing impacts on the family microsystem and its interfaces with community support systems that changed the routine of children and families in the initial moment of coping with the COVID-19 pandemic.

These changes, during the first seven months of the pandemic, particularly affected three axes of Nurturing Care (responsive care, learning and safety/protection), a World Health Organization framework aimed at supporting the global development of children. In pandemic situations, such as the COVID-19 pandemic, social isolation, stress, interruptions in family routine, stress and anxiety caused direct impacts on the full development of young children in their first years of life^(8)^.

The reality, which was already difficult, from financial (average of 1 to 1.5 minimum wages of family income in the population studied) to access to health (family health units only serving cases of greater urgency or related to COVID-19) and closure of day care centers and preschools, intensified parental relationships in the initial period of the COVID-19 pandemic, bringing additional demands, such as greater support from caregivers for their children’s school activities.

Family stressors such as unemployment and interpersonal difficulties, in addition to the scarcity of community resources, have a direct impact on child care and parenting^(16)^. The role of parents’ work in parental relationships is recognized as a requirement for stress at work or even the lack of it, implying the way adults deal with children in responsive or hostile interactions^(3)^.

Less direct interaction of children with support networks outside the family, such as the neighborhood and educational services, interferes with the cognitive development and socio-emotional development of children and their caregivers^(17)^. Difficulties in accessing school activities were identified for children from rural areas, poorer children with reduced access to technological devices and children whose caregivers live with a higher load of stress^(8)^.

Parental relationships during the COVID-19 pandemic and during the readaptation context are different^(18)^, depending on the location and subcultures, which were different from families with more material resources and in which the greater engagement of caregivers in reading and school activities was found^(8)^.

The school routine was one of the most affected according to reports from mothers, who, without the support of day care centers and schools, during the most critical period of the pandemic, were unable to effectively support pedagogical activities with children, both due to overload of domestic activities and access to technologies. This point was considered one of the main aggravating factors regarding parental stress and child development^(18, 19)^.

With regard to parenting practices, it was possible to notice mainly the mothers’ pleasure in playing and taking care of their children with playful activities. Caregivers also shared reports of praise and positive parenting practices, such as participation in games and affection in parental relationships. For positive and cognitively stimulating family environments, one of the most important was the presence of parents with their children in caring for and playing with children. Positive environments can have an impact on the development of these children with an effect on parents’ mental health as well^(20, 21)^.

In this interaction of play and care, negative parenting practices were also reported, such as punishment and physical violence.

Increased intra-family tension became potential for violence, including harsh discipline, neglectful parenting, and child abuse. The increase in negative parenting practices in the context of the COVID-19 pandemic was reported from the increase in situations such as screaming and spanking by caregivers as a form of discipline. These high rates may be related both to existing cultural practices, as well as to greater stress and burden on caregivers^(6)^.

In our study, fathers’ participation was perceived mainly through support for caregivers in household chores. Considering that half of the interviewees were single or did not live with their partners, the absence of affective support from parents in education and interaction with children in contexts of poverty deserve to be further addressed in interventions aimed at promoting child development and positive parenting.

There is relevance of the connection and need for environments such as homes, parents’ work, school to work together, according to the Bioecological Model assumptions^(3)^. The importance of the proximal environment and strong relationships between caregivers and children, for the moral, intellectual and socio-emotional development of children, must be strengthened by integrated interventions that understand the influence of mesosystem, exosystem and macrosystem variables, such as caregivers’ emotional well-being, access to the world of work, conditional income transfer and support from qualified support networks, such as health and education, with a focus on equity and minimization of negative effects for the full development of children living in poverty contexts^(17)^.

Interventions were health care-based with a focus on child development in low- and middle-income countries. An initiative in sub-Saharan Africa, encountered difficulties in incorporating caregivers’ knowledge about practices to promote child development aimed at learning opportunities less frequently and by about half of caregivers. This information leaves doubts whether mothers did not receive information about child development and parenting practices or whether they paid less attention to messages on the subject^(22)^.

In the time component, differences were perceived in the quantity and quality of time of mothers who did not have any work and who felt more overwhelmed during the period of the COVID-19 pandemic and mothers who worked, who recognized the need to have more time to interact with their children. In the historical perspective of time, all caregivers mentioned the desire for a better future for their children, based mainly on educational aspects, which would bring results for productive work.

The results found are in line with evidence on parenting in contexts prior to the COVID-9 pandemic, which highlighted that children who grow up in poverty have limited access to affective and learning resources that impact on adult life, including the parental role, with the reproduction of patterns, such as negative parenting practices. One of the explanatory models is the Family Stress Model, in which poverty associated with economic difficulties can lead to family stress and have a negative impact on parents’ emotional well-being and mental health. This aspect can influence parental behavior and increase the likelihood of harsh and controlling parenting practices. This model postulates that parents stressed and overwhelmed by the pressures of poverty may be unable to meet their children’s emotional, cognitive and caregiving needs^(23, 24)^.

### Study limitations

The limitations were the online data collection, making it impossible to apprehend non-verbal elements during interaction with caregivers and the presence of only mothers without the participation of fathers. We suggest that new studies be carried out in the future with a wider range of participants, including different profiles of primary caregivers on the subject in different cultures.

### Contributions to nursing and health

This work helps health professionals and professionals from other areas such as education and social assistance to reflect on parenting and child development in the context of the pandemic. The importance of diagnostic data to support future interventions is ratified.

## FINAL CONSIDERATIONS

Understanding the parenting practices experienced by the mothers in the study made it possible to identify emerging needs from the COVID-19 pandemic and possibilities for welcoming and forming a support network in the face of the demands encountered. Integrated interventions by health professionals, intersectoral actions and the closest community, can be factors that minimize future negative impacts for children who already lived in adversity contexts.

Interventions to support positive parenting become more urgent in the face of changes brought about by the COVID-19 pandemic in families living in a context of poverty. This perspective will be able to bring better results in global development for these children and minimize negative results, such as the limitations of children living in vulnerable contexts in accessing support networks that strengthen their cognitive and social development, such as day care centers and preschools.
